# Source apportionment, source-specific health risks, and control factors of heavy metals in water bodies of a typical karst basin in southwestern China

**DOI:** 10.1371/journal.pone.0309142

**Published:** 2024-08-23

**Authors:** Dijin Mu, Jianan Meng, Sangju Wang, Shizhen Xiao, Hao Wang, Xiangxuan Sun, Pan Wu

**Affiliations:** 1 School of Karst Science, Guizhou Normal University/State Engineering Technology Institute for Karst Desertification Control, Guiyang, Guizhou, China; 2 College of Resources and Environmental Engineering, Guizhou University, Guiyang, China; Ardakan University, ISLAMIC REPUBLIC OF IRAN

## Abstract

Studying the apportionment of source-specific health risks and control factors for heavy metal pollution in karst regions is crucial for prevention and management. A typical karst basin was chosen in this study to investigate the pollution characteristics of heavy metals, source-specific health risks, and control factors. The results indicate that during the rainy season, As, Cd, and Pb, as well as As during the dry season, were the primary elements responsible for water pollution in the watershed. Comparative analyses showed that the absolute principal component-multiple linear regression (APCS-MLR) model better identifies and quantifies the sources of heavy metals in karst basin waters. The analysis of health risks revealed that during the dry season, heavy metals in the basin posed a moderate cancer risk to adults (10^−4^ < total cancer risk (TCR) < 10^−3^), whereas during the rainy season, these heavy metals posed a non-cancer risk (total hazard index (THI) > 1) and a moderate to high cancer risk (10^−4^ < TCR < 10^−2^). The APCS-MLR model combined with the health risk analysis showed that Industrial waste discharge sources are the main contributors to the health of basin residents (29.39%-52.57%), making dry season As a non-cancer risk for basin residents, as well as rainy season As and Cd a non-cancer risk and a high cancer risk for basin residents. Therefore, reasonable planning for upstream industrial production should be developed, and priority should be given to monitoring and treating As and Cd pollution in water. Analyses also showed that input pathways, dilution effects, and hydrochemical characteristics may influence the spatial and temporal variability of heavy metals in the basin. The results provide essential information and significant reference for prioritising and managing the health risks associated with heavy metal pollution in water bodies in karst areas.

## 1. Introduction

Heavy metals in the aquatic environment have received global attention due to their toxicity and persistence and the health risks they pose [1, 2]. Sources of heavy metals in the aquatic environment include natural and anthropogenic sources, which include bedrock weathering, soil erosion, and atmospheric deposition [3–5] and anthropogenic sources, which include mining, metal smelting, industrial manufacturing, and agriculture [6–10]. Expanding industrial and agricultural production globally is the leading cause of heavy metal pollution in river ecosystems, which may pose severe risks to natural ecosystems and human health [11–13].

Karst landscapes cover 15.2% of ice-free land globally [14], with approximately 20%-25% of the population relying heavily or entirely on karst groundwater for drinking water [15]. However, heavy metal contamination in karst water is serious and increasing [16], posing a serious threat to the health of karst area residents. Previous studies have demonstrated that due to the high weathering rate of carbonate rocks [17, 18], heavy metal elements in carbonate rocks are released rapidly, making carbonate weathering one of the main sources of heavy metals in karst regions [19]. In addition, the special geological structure of karst regions can lead to strong interactions between surface water and groundwater [20, 21], which allows rapid transport of anthropogenically-added heavy metals in karst regions, further exacerbating the heavy metal loads in aquatic ecosystems in karst regions. Currently, research on water quality in karst basins primarily focuses on assessing and managing pollution in groundwater and springs and has made much progress [22, 23]. Various methods have been employed for pollution source apportionment in karst regions. Compared to traditional multivariate statistical methods, the absolute principal component scores-multiple linear regression (APCS-MLR) and the positive matrix factorisation (PMF) models not only quantitatively calculate the contribution of pollution sources but also do not require accurate source composition spectral information, making them more convenient and efficient for pollution source allocation [24]. However, previous studies in the karst watershed have rarely combined multiple parameters with metal elements, and most lack seasonal comparisons. Furthermore, due to the highly complex hydrological conditions in karst regions, there are limited reports on the source-specific heavy metals and their associated health risks in karstic water systems.

Southwestern China is one of the largest continuous karst regions in the world. Karst in the region is well developed with complicated underground water systems, many of which are used as important sources of drinking and production water, and the protection of water systems has received attention [25, 26]. Studies have shown that industrial-scale mineral excavation and refining in karst or non-karst areas of southwestern China have led to serious heavy metal pollution in the surrounding environment and downstream of rivers [27]. Since 2012, a large number of industrial enterprises have been introduced in some watersheds in the region, dominated by metallurgical industries, which may present a risk of pollution by Cr, Mn, Ni, Cu, Zn, As, Cd, Sb, Pb [28–30]. However, the issue of heavy metals in karst basin waters has been neglected for a long time. Therefore, there is an urgent need to investigate the impact of human activities on heavy metals in the waters of karst basins and the effects of heavy metal pollution on human health.

Amidst the growing global heavy metal pollution, there is a notable scarcity of research on heavy metal contamination in the karst basin with elevated geochemical backgrounds. Additionally, there is a lack of comprehensive reports on the health risks associated with source-specific pollution in karst regions. Based on this, this study investigated nine heavy metals (Cr, Mn, Ni, Cu, Zn, As, Cd, Sb, Pb) in a typical karst basin. The objectives of this study were to (1) resolve the possible sources and contributions of heavy metals in the watershed by using multivariate statistical analysis and receptor models; (2) evaluate the human health risk of heavy metals from each source using health risk assessment models; and (3) determine the spatial and temporal distribution of dissolved heavy metals in karst watersheds and the possible controlling factors. The results of the study can provide important information to ensure the safety of drinking water for the local residents and the balance of the ecological environment and contribute to a better understanding of the geochemical behaviour of heavy metal elements in karst areas, which is of great value for the prevention and control of heavy metal pollution in karst areas.

## 2. Materials and methods

### 2.1. Study area

**Fig 1:**
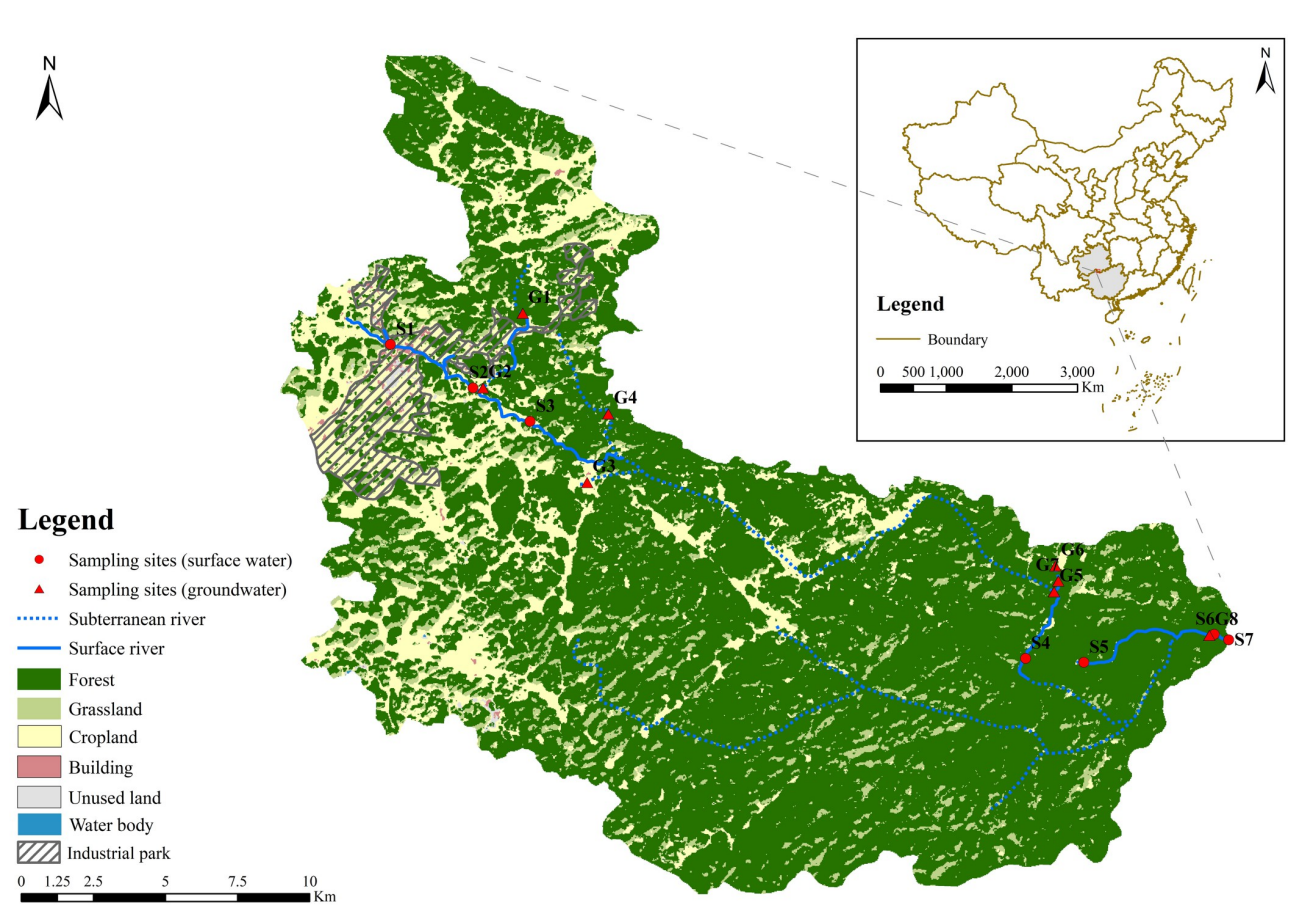
Map illustrating the study area and the positions of sampling sites. Notably, S1-S3 and G1-G4 represent sampling sites upstream, while S4-S7 and G5-G8 denote sampling sites downstream. The land cover mapping results from Yang and Huang’s work in 2021 [31].

The research site constitutes a representative karst basin in Dushan and its proximate regions in the southern Guizhou Province, Southwest China (Fig 1) [26]. The basin belongs to the subtropical monsoon humid climate zone, with an average annual temperature of 16.5°C and an average annual rainfall of 1,313 mm. The study area encompasses a catchment area of approximately 460 km^2^. The mainstream within the area has a total length of 56.5 km. The wet season, which spans from April to September, accounts for 84% of the total annual precipitation in the watershed. The maximum flow rate at the watershed’s outlet can reach 70 m^3^/s. In contrast, the dry season, which occurs from October to next March, experiences a minimum flow rate of 1.20 m^3^/s. The topography of the watershed is generally high in the north and low in the south, high in the east and low in the west, with rivers flowing from west to east.

The urban land in the watershed is mainly located upstream, and about 9.46 km^2^ was planned in 2011 to construct an industrial park mainly engaged in the metallurgical industry. Cultivated land in the basin is mainly located in the upper reaches, and most forest land is in the lower reaches.

### 2.2. Sample collection and analysis

According to field and historical data surveys, no access points to the underground river were found in the watershed’s southern tributaries and middle streams. Based on this, two sampling campaigns were conducted in the mainstream and selected tributaries of the basin during the dry and wet seasons (sampling dates: February and May 2022). The sampling encompassed seven surface water sites and eight groundwater sites (Fig 1), with a single water sample obtained from each site. Water temperature (WT), pH, dissolved oxygen (DO), and electrical conductivity (EC) were measured in situ using a multiparameter water quality analyser (ODEON Multy8320, Ponsel, France) with accuracies of 0.1°C, 0.01, 0.01 mg/L, and 1 μS/cm, respectively. HCO_3_^−^ and Ca^2+^ concentrations were measured in situ using an alkalinity test kit and a Calcium test kit (Merck, Germany), respectively. Titrations were conducted with an error margin of 0.1 mmol/L and 2 mg/L, respectively.

Water samples were collected approximately 50 cm below the water surface using a clean sampler, filtered through a 0.45 μm Millipore membrane filter, and then transferred to acid-washed and field water sample rinsed high-density polyethene (HDPE) bottles. Samples for cation and heavy metal analysis were acidified to pH < 2 using ultra-purified HNO_3_. All samples were stored in a portable holding tank and, if possible, returned to the laboratory within 24 hours.

Mg^2+^, Na^+^, K^+^, Cl^-^, SO_4_^2-^, PO_4_^3-^ and NO_3_^-^ were analyzed by ion chromatography (ICS-600, Thermo Fisher Scientific, USA) with accuracies of 0.02、0.02、0.02、0.007、0.018、0.051 and 0.016 mg/L, respectively. The concentrations of nine heavy metals (Cr, Mn, Ni, Cu, Zn, As, Cd, Sb, Pb) in the water samples were analyzed using an Inductively Coupled Plasma Mass Spectrometer (ICP-MS, Thermo Fisher Scientific, USA) under standard operating conditions. The detection limits for these metals were 0.11, 0.12, 0.06, 0.08, 0.67, 0.12, 0.05, 0.15, and 0.09 μg/L, respectively.

### 2.3. Multivariate statistical analyses

Heavy metal data were tested using Shapiro-Wilk (S-W) and MannWhitney U. Pearson’s correlation analysis was used to calculate the correlations between heavy metals as well as the heavy metals and water chemistry parameters, and weights were subsequently calculated. The water chemistry parameters significantly correlated with heavy metals at P < 0.05, as well as all the heavy metal parameters were selected to form the side and point files. These files were entered into Gephi to generate the network diagram [32]. Interpolation was used to represent the spatial distribution of heavy metal concentrations visually.

### 2.4. Heavy metal source apportionment

The APCS-MLR and the PMF are commonly used to identify pollutant source profiles and quantify their relative contributions, including the resolution of pollutant sources in water bodies such as surface water and groundwater [33–35]. Specific calculations for the APCS-MLR model are described in [36]. The PMF model was carried out using PMF5.0, downloaded from the U.S. EPA homepage.

After determining the appropriate model for source apportionment, the concentrations of source-specific heavy metals were computed using Eq (1), as presented by [37].


$$C_{ij}^{k}=C_{ij}^{k*}\times C_{ij}$$ (1)

Where $C_{ij}^{k}$ represents the concentration of the jth heavy metal from the kth source in the ith water sample (μg/L); $C_{ij}^{k*}$ denotes the contribution of the kth source to the jth heavy metal in the ith water sample; C_ij_ refers to the concentration of the jth heavy metal in the ith water sample (μg/L).

### 2.5. Health risk evaluation models

Several studies have indicated that heavy metals in the aquatic environment can potentially endanger human health through direct ingestion and dermal absorption [38–40]. In this study, a health risk assessment model was used to calculate the health risks of heavy metals in water bodies to adults and children through two exposure pathways [37, 41]. The specific calculation process is as follows:

$${CDI}_{{ij}_{ing}}^{k}=\left(C_{ij}^{k}\times IR\times {EF}_{ing}\times {ED}_{ing}\right)/\left(BW\times AT\right)$$ (2)

$${CDI}_{{ij}_{derm}}^{k}=\left(C_{ij}^{k}\times SA\times K_{p}\times ET\times {EF}_{derm}\times {ED}_{derm}\times 10^{-3}\right)/\left(BW\times AT\right)$$ (3)

$$HI={CDI}_{{ij}_{ing}}^{k}/{RfD}_{ing}+{CDI}_{{ij}_{derm}}^{k}/{RfD}_{derm}$$ (4)

THI=∑HI (5)

$$CR={CDI}_{{ij}_{ing}}^{k}\times {SF}_{ing}+{CDI}_{{ij}_{derm}}^{k}\times {SF}_{derm}$$ (6)

TCR=∑CR (7)

The variables ${CDI}_{ijing}^{k}$ and ${CDI}_{ijderm}^{k}$ represent the average daily intake of the jth heavy metal from the kth source at the ith sampling point through direct intake and dermal absorption routes, respectively. HI and THI denote the hazard index and total hazard index, respectively. The exposure reference dose for the corresponding heavy metal through direct intake and dermal exposure is represented by RfD_ing_ and RfD_derm_ (mg/kg/day). CR and TCR refer to cancer risk and total cancer risk, respectively. SF_ing_ and SF_derm_ ((mg/kg/day)^-1^) represent the slope factors for the corresponding heavy metals through direct intake and dermal absorption, respectively. The specific values for each parameter can be found in S1 and S2 Tables.

If both the HI and THI are below 1, there is no potential non-cancer risk; however, if either the HI or THI exceeds 1, there is a non-cancer risk and should be a cause for concern [37]. When CR and TCR are ≤ 10^−6^, human health has very low cancer risk; there is a low cancer risk when 10^−6^ < CR and TCR ≤ 10^−4^; there is a moderate cancer risk when 10^−4^ < CR and TCR ≤ 10^−3^; there is a high cancer risk when 10^−3^ < CR and TCR ≤ 0.1; there is a very high cancer risk when CR and TCR are > 0.1 [42].

## 3. Results and discussion

### 3.1. Characteristics of heavy metals and main parameters in the water bodies in the karst basin

Table 1 shows that PO_4_^3-^ was not detected in either season. The S-W test results indicated that during the dry season, the data for all parameters were normally distributed (values > 0.05) except for Cr, Mn, Ni, Zn, As, Cd, Pb, Na^+^ and SO_4_^2-^. In the wet season, the data for Mn, Mg^2+^, SO_4_^2-^, HCO_3_^-^, pH and DO were normally distributed (values > 0.05). Furthermore, the S-W test results, coefficient of variation (CV), and standard deviation (SD) suggested that outliers might influence the mean values of certain parameters [3]. Hence, median values of the concentrations were utilised for the analysis in this study.

**Table 1:** Statistics of heavy metal elements and physicochemical parameters in dry and wet seasons of the basin.

Parameters	Dry Season									Wet Season									MannWhitney U
	Min	Max	Mean	Meaden	SD	CV	Sk	K	S-W	Min	Max	Mean	Meaden	SD	CV	Sk	K	S-W	P
Cr(μg/L)	2.09	5.92	3.18	2.81	1.24	0.39	1.65	1.76	0.001**	0.00	1.88	0.31	0.23	0.47	1.53	2.87	9.63	0.000**	0.000**
Mn(μg/L)	0.00	37.27	5.14	0.58	10.73	2.09	2.60	6.56	0.000**	0.00	79.37	24.57	20.89	22.31	0.91	1.28	1.54	0.056	0.008**
Ni(μg/L)	1.67	5.56	2.74	2.24	1.32	0.48	1.55	1.35	0.001**	1.92	3.00	2.26	2.21	0.26	0.12	1.57	3.88	0.037*	0.600
Cu(μg/L)	0.68	1.53	1.00	0.93	0.25	0.25	1.06	0.52	0.072	0.00	0.85	0. 10	0.00	0.27	2.62	2.46	4.77	0.000**	0.000**
Zn(μg/L)	0.00	19.09	4.50	2.05	5.88	1.31	1.47	1.71	0.005**	10.15	637.05	69.05	24.84	158.14	2.29	3.79	14.54	0.000**	0.000**
As(μg/L)	0.17	15.71	2.71	1.32	4.11	1.51	2.78	8.57	0.000**	0.00	129.68	12.75	2.28	32.86	2.58	3.68	13.87	0.000**	0.570
Cd(μg/L)	0.03	1.26	0.29	0.17	0.34	1.18	2.08	4.92	0.002**	0.00	69.63	5.25	0.18	17.84	3.40	3.85	14.88	0.000**	0.570
Sb(μg/L)	0.19	1.00	0.44	0.38	0.23	0.53	1.08	1.07	0.269	0.00	2.68	0.77	0.33	0.89	1.16	1.54	1.21	0.001**	0.827
Pb(μg/L)	0.32	1.73	0.74	0.53	0.44	0.60	1.24	0.49	0.012*	0.03	24.79	3.39	1.32	6.24	1.84	3.28	11.51	0.000**	0.097
K^+^(mg/L)	0.00	1.22	0.61	0.59	0.33	0.55	0.49	0.15	0.626	0.00	1.08	0.24	0.17	0.32	1.34	2.00	3.40	0.000**	0.002**
Na^+^(mg/L)	0.04	1.00	0.43	0.37	0.36	0.83	0.47	-1.36	0.032*	0.00	0.19	0.01	0.00	0.05	3.87	3.87	15.00	0.000**	0.000**
Ca^2+^(mg/L)	68	86	78	76	5	0.07	-0.53	0.25	0.491	68	84	73	72	4	0.06	1.40	2.71	0.050*	0.035*
Mg^2+^(mg/L)	1.66	4.19	2.93	2.70	0.86	0.29	-0.20	-1.17	0.415	0.20	2.27	0.86	0.78	0.58	0.68	0.98	0.94	0.158	0.000**
Cl^-^(mg/L)	1.192	2.306	1.650	1.629	0.344	0.21	-0.20	0.88	0.548	0.000	4.460	1.333	0.564	1.493	1.12	1.20	-0.15	0.001**	0.045*
NO_3_^-^(mg/L)	0.000	2.990	1.587	1.678	0.800	0.50	0.08	-0.66	0.971	0.420	12.800	3.474	2.885	3.060	0.88	2.20	6.08	0.003**	0.018*
PO_4_^3-^(mg/L)	ND	ND	ND	ND	ND	ND	ND	ND	ND	ND	ND	ND	ND	ND	ND			ND	ND
SO_4_^2-^(mg/L)	4.333	7.738	6.217	6.433	0.952	0.15	-1.96	4.82	0.007**	1.660	12.860	8.024	7.539	2.549	0.32	-0.50	2.34	0.135	0.001**
HCO_3_^-^(mg/L)	201.4	231.9	218.8	216.6	8.9	0.04	-0.82	1.15	0.098	189.2	238.0	209.1	207.5	15.4	0.07	0.64	-0.20	0.197	0.101
WT(°C)	9.2	17.4	14.8	15.2	2.4	0.16	-0.73	0.59	0.143	14.8	20.0	18.4	18.6	1.3	0.07	-1.50	3.52	0.047*	0.000**
pH	8.08	8.91	8.37	8.39	0.25	0.03	0.42	-0.69	0.513	7.43	8.25	7.80	7.74	0.26	0.03	0.27	-0.92	0.624	0.000**
DO(mg/L)	7.60	10.52	8.98	9.04	0.88	0.10	0.11	-0.36	0.794	5.85	9.69	8.33	8.30	1.03	0.12	-0.74	0.88	0.317	0.085
EC(μS/cm)	283	388	325	318	29	0.09	0.59	0.14	0.703	316	430	345	333	32	0.09	1.54	2.26	0.008**	0.029*

Sk: skewness; K: kurtosis; ND: not detectable; Significance codes

***, <0.001

**, 0.001~0.01

*, 0.01~0.05.

All water bodies in the basin exhibited a weakly alkaline nature, with the median pH being lower during the wet season (7.74) compared to the dry season (8.39). The median EC was also lower in the dry season (318 μs/cm) than that in the wet season (333 μs/cm). This pattern is consistent with the characteristics of water bodies in numerous karst regions, as observed in previous studies [16, 43, 44]. The median WT was 15.18°C and 18.58°C in the dry and wet seasons, respectively, and the median DO was 9.04 mg/L and 8.30 mg/L, respectively, which showed a decrease in dissolved oxygen with increasing temperature [45]. In terms of cations, the median values in both seasons followed the pattern of Ca^2+^ > Mg^2+^ > K^+^ > Na^+^. Similarly, the medians of the four anions were observed to be HCO_3_^-^ > SO_4_^2-^ > NO_3_^-^ > Cl^-^. Apparently, the major anions and cations in the watershed are HCO_3_^-^ and Ca^2+^, respectively, influenced by the widely distributed carbonate rocks in the watershed, and the water chemistry type of the watershed is HCO_3_-Ca [43].

Regarding heavy metals, the median values during the dry season followed the sequence of Cr > Ni > Zn > As > Cu > Mn > Pb > Sb > Cd. Conversely, during the wet season, the sequence was Zn > Mn > As > Ni > Pb > Sb > Cr > Cd > Cu. The notable variation in the median heavy metal concentration sequences between the two periods may be attributed to different sources for each metal during the respective periods or varying contributions from the same source to the heavy metals [3].

**Fig 2:**
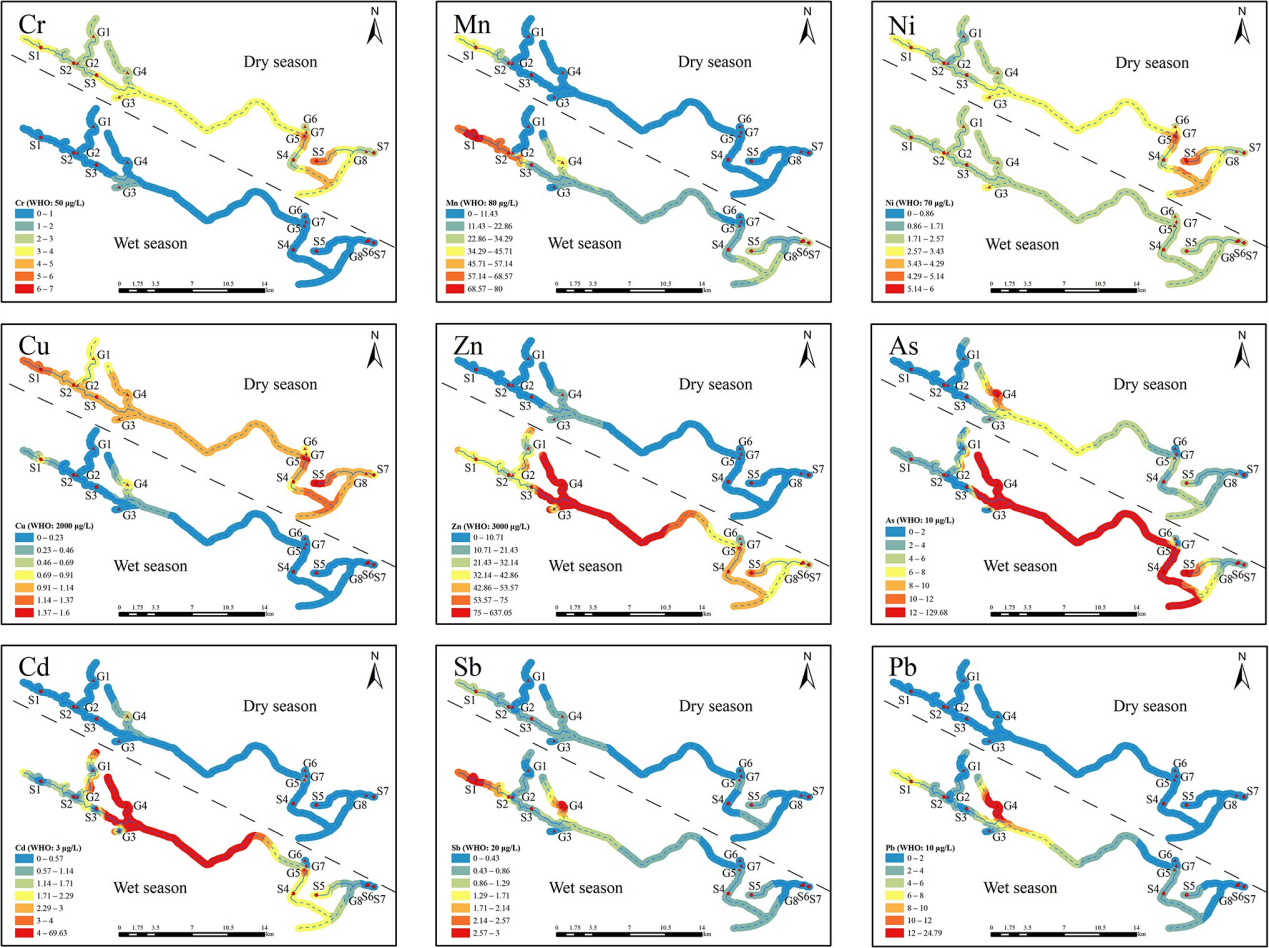
Seasonal and spatial variations in the concentrations of nine heavy metal elements in the basin. In this figure, “WHO” denotes the standard limits for heavy metals as provided by the WHO.

Compared to the World Health Organization (WHO) standard (Fig 2), the median values for the respective parameters in the basin were lower than the standard limits. This indicates that the overall water quality of the basin’s waters was good. However, it is important to note that during the wet season, the concentrations of As, Cd, and Pb in the upstream G4 water samples exceeded the corresponding standard limits. Additionally, the concentrations of As in the downstream G5, S4, and S5 water samples and Cd in the G5 water samples were higher than the standard limits. Similarly, during the dry season, the concentration of As in the G4 water samples exceeded the standard limits. Based on these findings, it can be concluded that As, Cd, and Pb are the main heavy metal elements contributing to water pollution in the karst watershed. Notably, in karst areas with high geochemical background, the concentrations of most heavy metals are high, especially As and Cd [46].

### 3.2. Spatial and temporal variation of heavy metals in the karst basin

The results of the MannWhitney U test revealed that there were significant seasonal variations (p < 0.05) in the observed values of Cr, Mn, Cu, and Zn, whereas the observed values of Ni, As, Cd, Sb, and Pb showed insignificant seasonal variations (Table 1). In the dry season, the median concentrations of Cr, Ni, Cu, and Sb were higher compared to the wet season, while the median concentrations of the remaining metals were lower. Interestingly, the concentrations of Cr and Cu were higher in all monitoring points during the dry season than those in the corresponding wet season. This seasonal variation could be attributed to rainfall wet deposition, scouring, leaching, and dilution, which can alter the sources, pathways, and concentrations of heavy metals entering the basin waters [47].

Previous research has demonstrated a strong connection between surface water and groundwater in the basin [26]. In this study, the concentrations of heavy metals in surface water and groundwater were similar during the dry season, excluding Mn, and during the wet season, excluding Mn, Zn, As, and Pb. This indicates that most metals in the basin are influenced by the connectivity between surface water and groundwater, resulting in minimal differences in the distribution of heavy metal concentrations [20]. Consequently, a separate analysis of the distribution of heavy metals in surface water and groundwater was not conducted in this study.

As shown in Fig 2, in the dry season, the concentrations of all metals, except for Cr, Ni, and Cu, were usually higher in the upstream compared to the downstream. However, for Cr, Ni, and Cu, the concentrations were slightly elevated downstream compared to upstream. In the wet season, all metals usually exhibited higher concentrations in the upstream than the downstream, with the concentration of Cr upstream nearly equivalent to that downstream. Hence, it can be inferred that Cr, Ni, and Cu in the dry season and Cr in the wet season are subjected to fewer influences from human activities. In contrast, the remaining heavy metals in both seasons are more impacted by human activities.

The concentrations of Mn, Sb, and Pb in S1 and S2, as well as Zn, As, Cd, Sb, and Pb in G4, were significantly higher in both periods. Specifically, during the wet season, the concentrations of Zn, As, and Cd in G4 were notably elevated compared to those in the other samples. Clearly, S1, S2, and G4 exhibit significant impacts from human activities.

### 3.3. Apportionment of heavy metal sources in the karst basin

#### 3.3.1. Comparison of heavy metal source apportionment between APCS-MLR and PMF models

**Fig 3:**
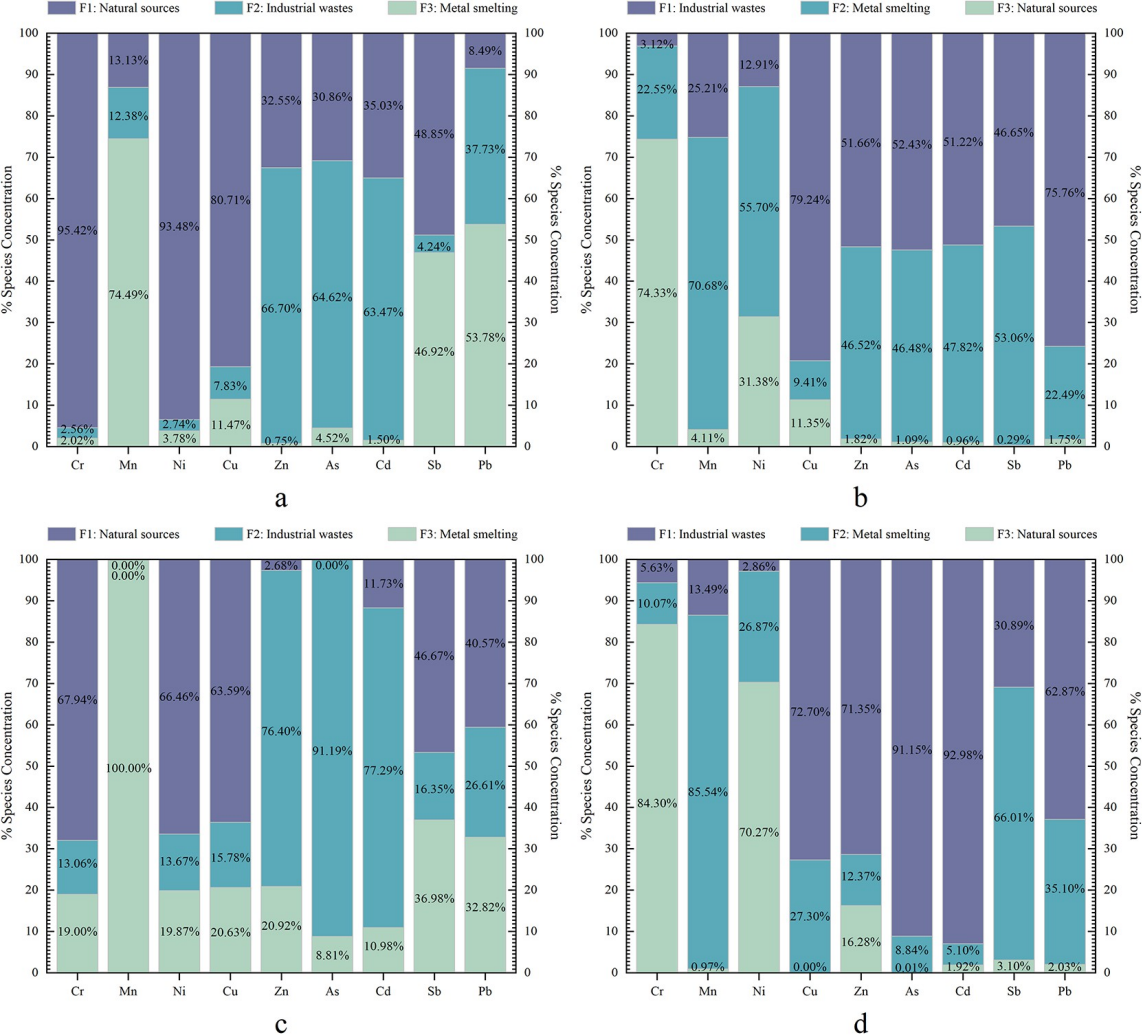
Contribution of APCS-MLR model (a: dry season; b wet season) and PMF model (c: dry season; d wet season) extraction factors to heavy metals.

As depicted in S1 Fig, the APCS-MLR model exhibited satisfactory results, with an R^2^ greater than 0.5 for the nine heavy metals in both periods. The mean R^2^ value was 0.900 for the dry season and 0.930 for the wet season, indicating a good fit. Fig 3A and 3B show that the APCS-MLR model extracted three factors in both periods. In the dry season, factor 1 contributed 95.42%, 93.48%, and 80.71% to Cr, Ni, and Cu, respectively. Factor 2 accounted for 66.70%, 64.62%, and 63.47% of Zn, As, and Cd, respectively. Factor 3 contributed 74.49%, 46.92%, and 53.78% to Mn, Sb, and Pb, respectively. During the wet season, factor 1 contributed 79.24%, 51.66%, 52.43%, 51.22%, and 75.76% to Cu, Zn, As, Cd, and Pb, respectively. Factor 2 contributed 70.68%, 55.70%, and 53.06% to Mn, Ni, and Sb, respectively. Factor 3 contributed 74.33% to Cr.

Nine metals had a signal-to-noise ratio greater than one and were classified as "strong", which ensured the reasonableness of the PMF model. Three factors were identified by minimising the objective function Q in the PMF model, which further validated the results of the APCS-MLR model (Fig 3C and 3D). In the dry season, the PMF model yielded an average R^2^ value of 0.853, with R^2^ greater than 0.8 for all heavy metals except Zn (R^2^ = 0.257) (S2 Fig). In the wet season, the mean R^2^ value for the PMF model was 0.769, with R^2^ greater than 0.6 for all heavy metals except Cr (R^2^ = 0.239) and Ni (R^2^ = 0.129) (S3 Fig). During the dry season, factor 1 contributed over 60% to Cr, Ni, and Cu, accounting for 67.94%, 66.46%, and 63.58%, respectively. Factor 2 contributed 76.40%, 91.19%, and 77.29% to Zn, As, and Cd, respectively. Factor 3 contributed the most to Mn with 100%, followed by Sb and Pb with 36.98% and 32.82%, respectively. In the wet season, factor 1 had the higher contributions to Cu, Zn, As, Cd, and Pb, with percentages of 72.70%, 71.35%, 91.15%, 92.99%, and 62.87%, respectively. Factor 2 contributed 85.54%, 26.87%, and 66.01% to Mn, Ni, and Sb, respectively. Factor 3 made the most significant contribution to Cr, accounting for 84.26%, followed by 70.27% for Ni.

The above results indicate that the results of heavy metal source apportionment are similar for both models. However, the APCS-MLR model demonstrated higher R^2^ values compared to the PMF model for both periods(S1–S3 Figs). These findings suggest that the APCS-MLR model provides a better fit than the PMF model. Thus, in this study, the APCS-MLR model is deemed more suitable for allocating multiple heavy metal pollutant sources to water bodies in karst regions with complex hydrogeological structures.

#### 3.3.2. Identification of heavy metal sources in the karst basin

**Fig 4:**
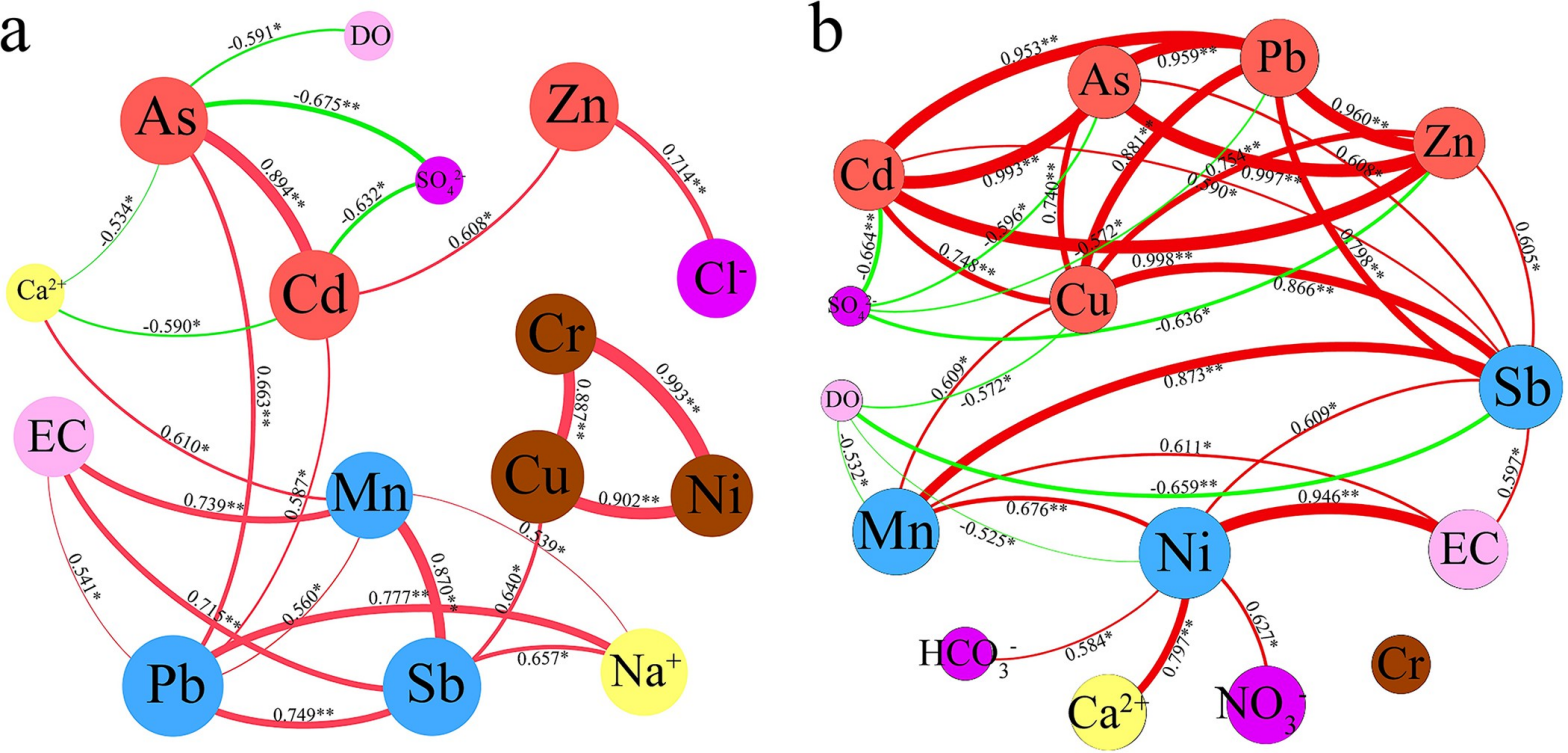
Co-occurrence network of water chemistry factors with heavy metals in dry (a) and wet (b) seasons. In the co-occurrence network, the size of the nodes is related to the value; green lines indicate negative correlations and red lines indicate positive correlations; line widths indicate the magnitude of the correlation coefficients; * indicates that the correlation is significant at the P < 0.05 level and ** indicates that the correlation is significant at the P < 0.01 level.

Fig 4 shows that during the dry season, the nine heavy metals can be divided into three groups based on the correlation between the heavy metals. There is a significant positive correlation between the heavy metals within each group, namely Cr, Ni, Cu; Mn, Sb, Pb; and As and Cd. In the wet season, all heavy metals, except for Cr, displayed a significant positive correlation with Sb. In addition, Cu, Zn, As, Cd, Pb, Mn, and Ni can be divided into two groups, and there is a significant positive correlation between heavy metals in each group. These findings suggest that heavy metals within these groups may have similar sources in both the dry and wet seasons (Xu et al., 2020). There is a difference in the correlation between heavy metals in the dry and wet seasons, probably because the same heavy elements have different significant sources in the two periods, consistent with the analyses in 3.1 and 3.2.

Previous studies have established that Cr, Ni, and Cu metal elements primarily originate from natural sources [48], such as weathering of bedrock or soil erosion [16, 49]. In our study, dry season Cr, Ni, and Cu had high loadings in Factor 1, and there was a positive correlation between them, indicating that they are of similar origin. Interestingly, the mean concentrations of Cr, Ni, and Cu during the dry season were slightly higher in the lower reaches of the watershed, where human activity was limited, compared to the upper reaches with more pronounced human activity. Additionally, the coefficients of variation (CV = 0.39, 0.48, and 0.25) for Cr, Ni, and Cu were relatively low, suggesting minimal variability of these elements in the basin’s waters. A separate investigation indicated comparable levels of Cr, Ni, and Cu in bedrock between the upper and lower parts of the basin and similar levels of these elements in soils in both regions [50]. These findings lead us to assume that factor 1 during the dry season predominantly reflects the influence of natural sources. During the wet season, the concentrations of Cr at all monitoring sites were lower than the minimum values observed during the dry season at the monitoring sites. Moreover, the average concentrations of Cr upstream and downstream during the wet season were nearly identical. This observation aligns with previous research, which demonstrated that although the dissolution of carbonate rocks in the watershed can increase the flux of certain metals in water during the wet season under natural conditions, the dominant effect is dilution, resulting in lower concentrations of these metals [43]. Hence, based on the study, it is reasonable to assume that the wet season Cr is primarily influenced by natural sources. Specifically, wet season factor 3 reflects the dominance of natural sources.

The coefficients of variation for Mn exhibited high values in both periods. Furthermore, the Mn concentrations in water samples obtained from S1 and S2 during both periods, as well as G4 during the wet season, were significantly higher compared to the other sampling sites. These findings strongly suggest that Mn in the watershed is influenced by human activities. Previous research has demonstrated that the metal industry serves as a primary contributor to elevated Mn concentrations [51]. In this study, many metal smelting industries, including Mn, Ni, and Sb, were observed, many of which were located near water sampling sites S1, S2, and G4 in the upper part of the basin. Consequently, it can be reasonably inferred that industries associated with Mn smelting play a crucial role in the presence of Mn within the water in the basin. Sb is similar to Mn, and the concentration of Sb in the water samples of S1 and S2 in both periods and G4 in the wet season was significantly higher than those in the other sampling sites. Moreover, it is well-documented that metals such as Ni and Pb are commonly released into the environment during metal smelting operations [30, 52]. Therefore, it is plausible to consider dry season factor 3 and wet season factor 2 as the primary sources of metal smelting emissions.

Fig 2 demonstrates that during the wet season, the concentrations of Zn, As, Cd, and Pb in water samples from G4 were significantly higher than the other monitoring sites. Additionally, Cu levels in both S1 and G4 water samples were significantly higher than those in the other monitoring sites. These metals are affected by human activities during the wet season. Similarly, the concentrations of Zn, As, and Cd in water samples from G4 were higher during the dry season, suggesting an impact of human activities on these metals. According to the survey, industrial enterprises in the upper part of the watershed generate a large amount of waste residues and liquids, and there are leakages. These industrial waste materials contain metals such as Zn, Pb, Cd, and As [28, 29, 53], which may enter the water bodies of the watershed through rainfall leaching or direct pathways. However, the transport pathways of metals may differ between the dry and wet seasons, as evidenced by a significant positive correlation between Cd distribution and As and Zn during the dry season and a significant positive correlation between Cu, Zn, As, Cd, and Pb during the wet season. Based on the results of PCA, it is reasonable to assume that dry season factor 2 and wet season factor 1 represent industrial waste-related factors.

### 3.4. Health risks from specific sources of heavy metals in the karst basin

**Fig 5:**
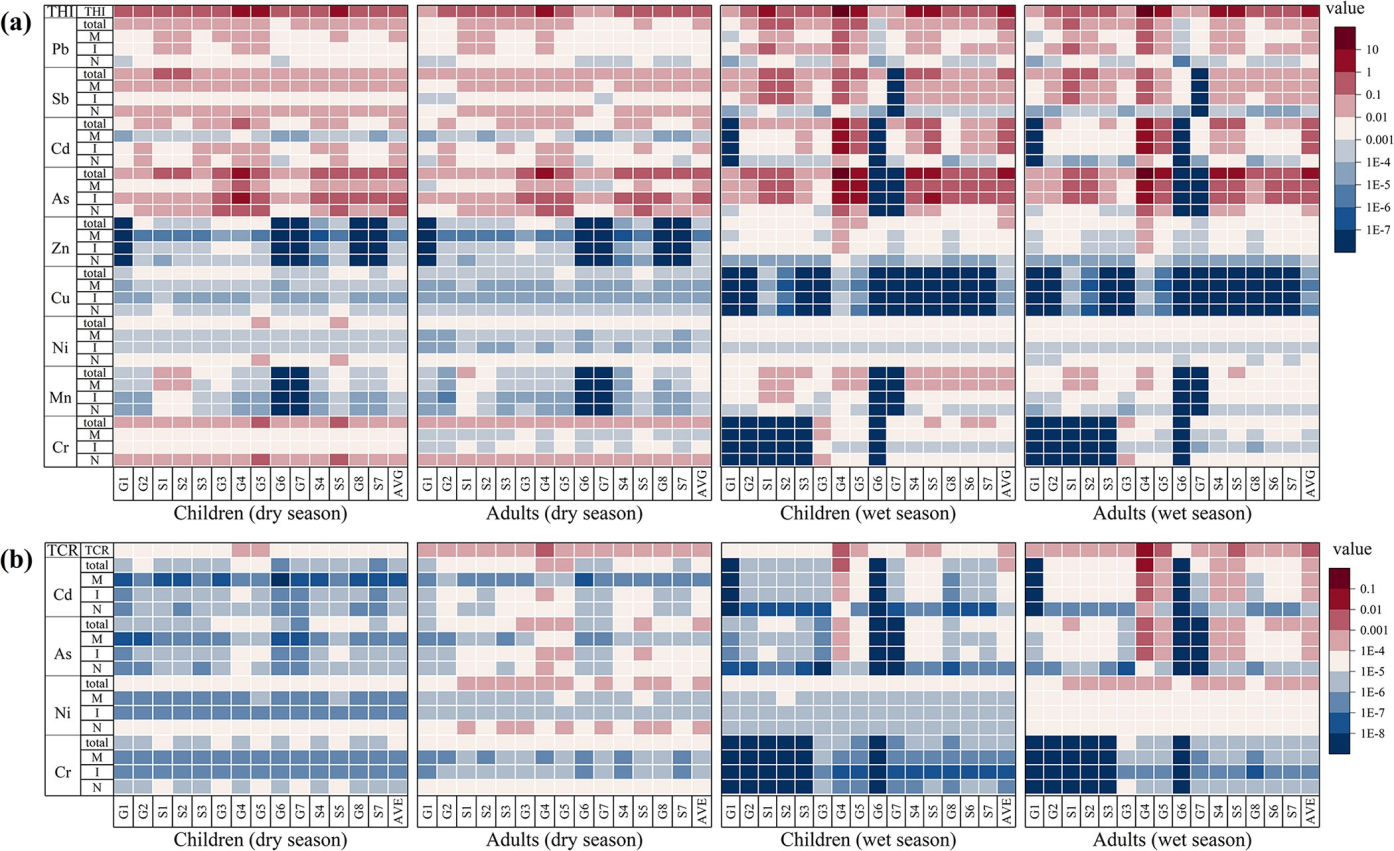
Non-cancer (a) and cancer (b) risk of source-specific heavy metals at each sampling site. N is for natural sources; I is for sources of industrial waste; and M is for sources of metal smelting.

Regarding non-cancer risk (Fig 5A), the mean THI values for children and adults in the basin during the dry season were below 1, indicating no potential non-cancer risk of metals in the basin at that time. However, particular areas in the basin exhibited non-cancer risk to humans. For example, the THI values for children and adults in water body G4 and children in water bodies G5 and S5 exceeded 1. During the wet season, the mean THI values for children and adults in the watershed exceeded 1, indicating a non-cancer risk for the entire watershed. Additionally, the presence of higher non-cancer risk in specific areas of the watershed was evident, with higher non-cancer risk observed for children in water bodies S1, G4, G5, S4, and S5, and for adults in water bodies G4, G5, S4, and S5. Water body G4 exhibited a high non-cancer risk for children (THI = 27.07) and adults (THI = 17.62). It is worth noting that children’s THI and HI values in both periods were higher than those of adults, consistent with previous studies [54, 55]. This can be primarily attributed to children’s higher water consumption per unit of body weight and increased vulnerability to environmental factors and pollutant absorption, rendering them more prone to non-cancer risks [56].

Regarding cancer risk (Fig 5B)., the mean TCR values for children in the dry season basin ranged from 10^−5^ to 10^−4^, suggesting a low cancer risk posed by heavy metals during this season. However, the mean TCR values for adults in the dry season ranged from 10^−4^ to 10^−3^, indicating a moderate cancer risk from heavy metals for adults in the basin. In the wet season, the mean TCR values for children ranged from 10^−4^ to 10^−3^, indicating a moderate cancer risk. On the other hand, the mean TCR values for adults ranged from 10^−3^ to 10^−2^, indicating a high cancer risk during this season. Overall, the mean TCR values were higher in the wet season than that in the dry season, suggesting a greater threat to human health during the wet season. Similar to the non-cancer risk, the highest TCR values were observed in G4 in both the dry and wet seasons, particularly in the wet season, where the water in G4 posed a high cancer risk to children and adults. Notably, TCR and CR values were higher in adults than those in children, which aligns with previous research [57, 58]. This pattern can be attributed to the longer exposure of the adult population to heavy metal pollutants and the subsequent accumulation of more carcinogens in their bodies, thereby increasing their risk of developing cancer.

It can be seen in Fig 5 that there is a large variation in the human health risk associated with different types of heavy metals and a large variation in the contribution of the type of heavy metal source to the human health risk. During the dry season, children and adults are primarily exposed to non-cancer risks from As, Cr, Sb, Cd, and Pb, while cancer risks mainly arise from Ni, As, and Cr. Among these, the health risks associated with As for residents in the watershed are primarily attributed to industrial waste emissions, leading to non-cancer risks (HI > 1) for the population in the watershed. It is worth noting that high concentrations of arsenic can lead to diabetes, hepatitis, cancer, cardiovascular diseases, and haematological disorders [59, 60]. During the rainy season, non-cancer risks primarily originate from As, Cd, Pb, and Sb, while cancer risks primarily stem from Cd, As, and Ni. Industrial waste emissions are the main contributing factor to the impacts of Cd and As on human health during the rainy season. As a result, both non-cancer risks (HI > 1) and high cancer risks (CR > 10^−3^) exist for children and adults exposed to As and Cd. Long-term consumption and exposure to water containing high levels of As and Cd can negatively affect human health [61–63]. In summary, industrial activities are the main factors affecting the health of residents in the watershed. Therefore, it is essential to develop rational plans for upstream industrial production and regularly monitor the levels of As and Cd in water, with priority given to addressing areas with severe As and Cd contamination.

**Fig 6:**
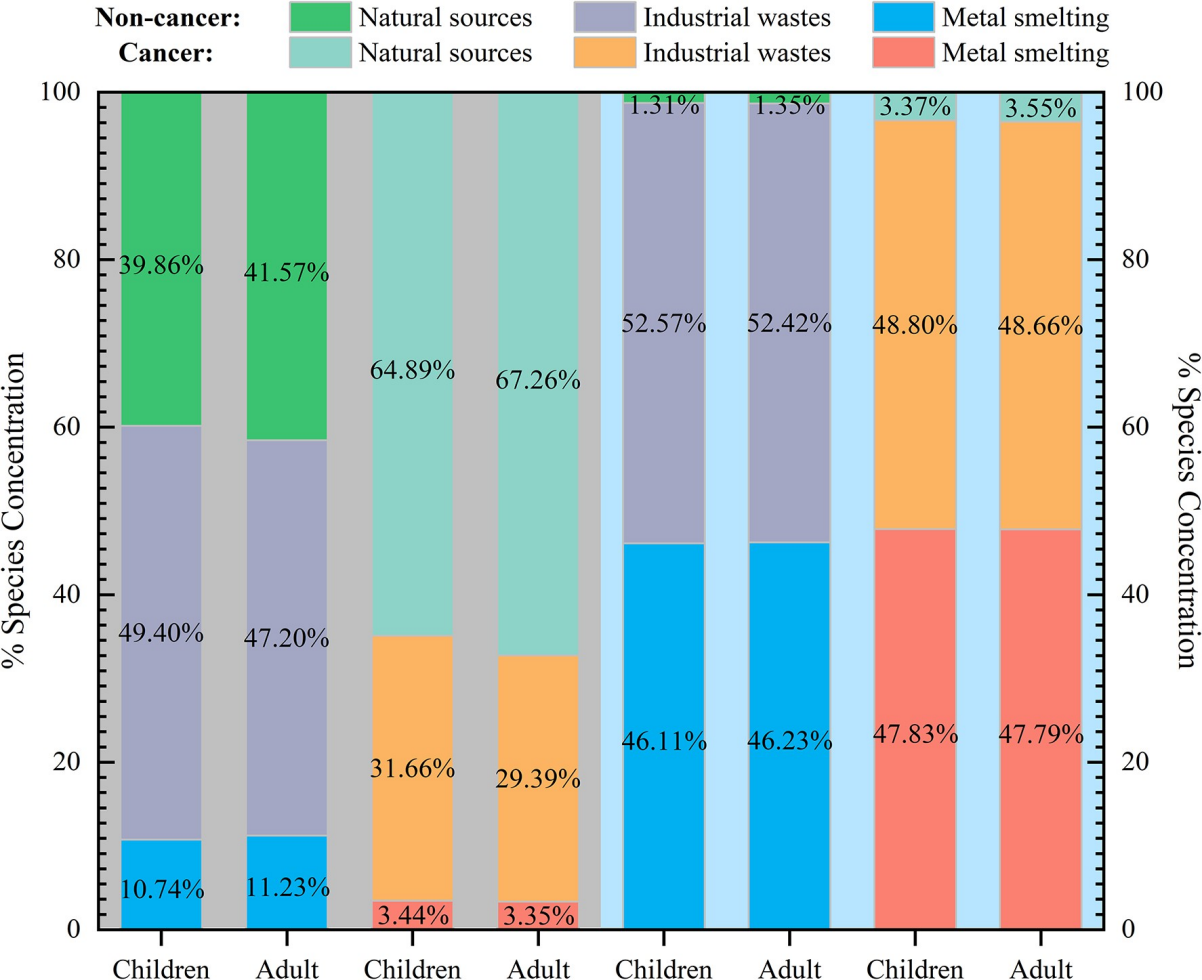
The proportion of source-specific contributions to human health risk in the dry (left) and wet (right) seasons.

The contribution of different sources to the non-cancer risk and cancer risk associated with the nine heavy metals exhibited variability, as illustrated in Fig 6. During the dry season, industrial waste emission sources (49.40%, 47.20%) and natural sources (39.86%, 41.57%) were identified as the primary contributors to the non-cancer risk for children and adults. The cancer risk for children and adults during the dry season was predominantly attributed to natural sources (64.89%, 67.26%). In contrast, the non-cancer and cancer risks for children and adults during the wet season were most significantly influenced by industrial waste emission sources (52.57%, 52.42%; 48.80%, 48.66%), followed by emissions from metal smelting sources (46.11%, 46.23%; 47.83%, 47.79%).

### 3.5. Factors controlling heavy metals in the karst basin

Differences in the types of sources directly influence the distribution of heavy metals within the watershed. Furthermore, the pathways through which heavy metals are introduced from various sources can also significantly influence their concentrations. In this study, for instance, the higher concentrations of Mn and Sb observed in S1 and S2 during the dry season can be attributed to the direct deposition of soot containing these metals from the metal smelting industry into the surface water. On the other hand, the soil above the groundwater in G4 intercepts this soot, preventing its direct entry into the groundwater. However, during the wet season, the soot is washed into the groundwater due to rainfall, leading to higher concentrations of Mn and Sb in this season.

It is important to highlight that the phenomenon of dilution can play a significant role in explaining the seasonal variations observed in certain heavy metals. For instance, in both the dry and wet seasons, the majority of Cr present was found to be of natural origin (dry season: 95.42%, wet season: 74.33%). However, despite this similarity, the concentration of Cr in the wet season was significantly lower than that in the dry season due to the effects of dilution. The limited contribution of Cr from natural sources during the wet season was diluted, resulting in reduced concentrations [43]. Similarly, even though human activities primarily influenced the concentration of Cu in the wet season (88.65%), the limited input of Cu into the river could not overcome the dilution effect, leading to a significantly lower concentration than in the dry season.

Previous research has established that wastewater or leachate from various industrial activities such as mining, metallurgy, and coal-related processes is typically acidic [64, 65]. The fast erosion process of carbonate rock in karst areas causes the high HCO_3_^−^ concentrations and pH in karst water body [66, 67]. Owing to the wide prevalence of carbonate minerals in karst areas, this alkaline milieu exhibits high stability, facilitating the swift neutralization of acidic waters containing high metal loads and facilitating the precipitation of certain metals (e.g., Cd, Pb, Zn) through the formation of insoluble species [32, 68]. In this study, the water samples collected from all monitoring points within the basin during both periods exhibited alkaline properties (see Table 1), even though certain monitoring sites were significantly affected by discharges from metal smelting and industrial waste sources. Consequently, pH may be a significant influencing factor for some metals.

Fig 4 illustrates that multiple water chemistry parameters in karst water potentially affect the distribution of metals within the watershed. Previous research has indicated that in karst areas, the presence of SO_4_^2-^ leads to the production of carbonic acid, even in the absence of other sources of CO_2_ [69]. This process enhances the dissolution of carbonate rock, consequently increasing the release of metals into the water. Additionally, the abundance of SO_4_^2-^ in karst water facilitates the formation of complexes with certain metal ions, thereby reducing the concentration of free metal ions in the water [32]. However, in this study, the SO_4_^2-^ concentration was low, limiting its ability to increase metal inputs through carbonate dissolution. Instead, it potentially formed complexes with metals such as Zn, Cd, and Pb, gradually decreasing these metals from upstream to downstream. Unlike other metals, As can form complexes with the abundant Ca^2+^ in karst water, leading to its subsequent adsorption onto sediment surfaces. In addition, As can be adsorbed by precipitates formed by SO_4_^2-^ with other ions, thus reducing its concentration in water [67].

The concentration of dissolved DO can potentially influence the transportation of heavy metals in water bodies. In this study, the higher concentration of DO in the watershed waters may lead to the oxidation of certain metals, such as Fe and Mn. This, coupled with the alkaline nature of the watershed waters, results in the formation of water-insoluble compounds of these metals. These compounds can potentially adsorb some heavy metals and subsequently settle, reducing the overall concentration of heavy metals in the water body [70, 71]. EC represents the cumulative conductivities of various ions in a solution, indicating soluble salt concentration in aqueous solutions. As depicted in Fig 4, EC exhibited a significant correlation with Mn, Sb, and Pb during the dry season and with Mn, Ni, and Sb during the wet season. This indicates that human activities could potentially contribute to the increased presence of metal ions in the water, leading to an elevation in EC.

## 4. Conclusion

Currently, limited research has been conducted on the contamination of heavy metals in karst basins characterized by high geochemical backgrounds, and there is a dearth of literature addressing the health risks associated with heavy metals from source-specific in karst regions. In this study, a typical karst basin was selected as the research subject to analyse the pollution characteristics of heavy metals in the water bodies of the basin, as well as the potential control factors and source-specific health risks. The findings demonstrate that, in the rainy season, As, Cd, and Pb were the primary pollutants in the basin, with As predominant during the dry season. Based on the analysis, employing the APCS-MLR model made it possible to identify and quantify potential sources of heavy metals in karst basin waters more effectively. This study identified three main sources of metals in the basin in both seasons: natural sources, industrial waste emission sources, and metal smelting emission sources. During the dry season, industrial waste discharge sources and natural sources were the main contributors to both non-cancer and cancer risks for children and adults. Conversely, during the wet season, the non-cancer and cancer risks for children and adults were primarily associated with industrial waste discharge sources and metal smelting emission sources. The heavy metals present in the basin during the dry season posed a moderate cancer risk to adults, while those present during the wet season posed a non-cancer risk to all individuals and a moderate to high cancer risk to children and adults. The investigation reveals that industrial activities contribute to 58.43%-98.68% of the non-cancer risk and 32.74%-96.63% of the cancer risk. Therefore, effective control of industrial activities in the basin is critical and urgent. Additionally, the study suggests that source types, input pathways, dilution effects, and water chemistry characteristics may significantly influence heavy metals’ spatial and temporal variability. These findings offer crucial insights for prioritizing and mitigating health risks associated with heavy metal contamination in water bodies within karst regions, holding substantial value for managing water pollution in such areas.

## Supporting information

S1 Table Parameters of the health risk evaluation model. (PDF)

S2 Table Parameter values for health risk evaluation for heavy metals. (PDF)

S1 Fig Scatter plot of predicted and observed heavy metal concentrations using the APCS-MLR model. (PDF)

S2 Fig Scatter plot of predicted and observed dry season heavy metal concentrations using the PMF model. (PDF)

S3 Fig Scatterplot of predicted and observed heavy metal concentrations during the wet season using the PMF model. (PDF)
