# Supplementary material for: Source apportionment, source-specific health risks, and control factors of heavy metals in water bodies of a typical karst basin in southwestern China
Source: PLoS One. 2024 Aug 23;19(8):e0309142. doi: 10.1371/journal.pone.0309142 (PMC11343453; doi:10.1371/journal.pone.0309142)
Supplement: S1 Table — (PDF) [file pone.0309142.s001.pdf]

Table S1. Parameters of the health risk evaluation model.

Parameters	Units	Value		References
		Children	Adults	
Ingestion Rate（IR）	L/day	0.64	2	[1]
Body Weight（BW）	kg	15	70	[1]
Exposure Frequency of Ingestion（EFing）	Days/year	365	365	[2]
Exposure Duration of Ingestion（EDing）	years	6	70	[2]
Exposure Frequency of Derm（EFderm）	Days/year	350	350	[1, 3]
Exposure Duration of Derm（EDderm）	years	6	30	[3]
Skin Surface Area Available for Contact（SA）	cm^2	6600	18000	[1]
Exposure Time（ET）	h/day	1	0.58	[1]
Averaging Time(*AT*) (Non-cancer risk)	days	ED × 365	ED × 365	[3]
Averaging Time(*AT*) (Cancer risk)	days	25550	25550	[3]

References:

1. Xiao J, Wang L, Deng L, Jin Z. Characteristics, sources, water quality and health risk assessment of trace elements in river water and well water in the Chinese Loess Plateau. Sci Total Environ. 2019;650(2):2004-2012.
2. Wu J, Lu J, Luo Y, Duan D, Zhang Z, Wen X, et al. An overview on the organic pollution around the Qinghai-Tibet plateau: The thought-provoking situation. Environ Int. 2016;97:264-272.
3. USEPA. Risk Assessment Guidance for Superfund Volume I: Human Health Evaluation Manual (Part E, Supplemental Guidance for Dermal Risk Assessment). Washington,DC: U.S. Environmental Protection Agency, 2004.
