# Supplementary material for: Source apportionment, source-specific health risks, and control factors of heavy metals in water bodies of a typical karst basin in southwestern China
Source: PLoS One. 2024 Aug 23;19(8):e0309142. doi: 10.1371/journal.pone.0309142 (PMC11343453; doi:10.1371/journal.pone.0309142)
Supplement: S2 Table — (PDF) [file pone.0309142.s002.pdf]

Table S2. Parameter values for health risk evaluation for heavy metals: reference dose of Ingestion (RfDing), reference dose for the dermal route of exposure (RfDderm), slope factor of Ingestion (SFing), slope factor for the dermal route of exposure (SFderm) and dermal permeability coefficient (Kp).

Element	K_p[1]	RfD_{ing}[2, 3]	RfD_{derm}[3]	SF_{ing}[4, 5]	SF_{derm}[4, 5]
RfDing[5, 6]	cm/h	mg/kg/day	mg/kg/day	$(mg/kg\text{-}day)^{-1}$	$(mg/kg\text{-}day)^{-1}$
RfDderm[6]	0.002	0.003	0.000075	0.5	20
SFing[7, 8]	0.001	0.0003	0.000285	1.5	3.66
SFderm[7, 8]	0.001	0.0005	0.000025	6.1	3.8
	0.0001	0.0014	0.00042		
	0.001	0.14	0.00096		
Cr	0.0002	0.02	0.0008	1.7	42.5
As	0.001	0.04	0.012		
Cd	0.0006	0.3	0.06		
Pb	0.001	0.0004	0.000008		

Mn

References:

1. USEPA. Regional Screening Level (RSL) Chemical-specific Parameters Supporting Table. Washington, DC: United States Environmental Protection Agency, 2022 2022.
2. USEPA. Regional Screening Level (RSL) Resident Tap Water Table (TR=1E-06, HQ=1). Washington, DC: United States Environmental Protection Agency, 2022 2022.
3. Peng H, Yang W, Ferrer ASN, Xiong S, Li X, Niu G, et al. Hydrochemical characteristics and

health risk assessment of groundwater in karst areas of southwest China: A case study of Bama, Guangxi. J Clean Prod. 2022;341:130872.

4. Sheng D, Meng X, Wen X, Wu J, Yu H, Wu M. Contamination characteristics, source identification, and source-specific health risks of heavy metal(loid)s in groundwater of an arid oasis region in Northwest China. Sci Total Environ. 2022;841:156733.

5. Chen G, Wang X, Wang R, Liu G. Health risk assessment of potentially harmful elements in subsidence water bodies using a Monte Carlo approach: An example from the Huainan coal mining area, China. Ecotox Environ Safe. 2019;171:737-745.
