# Supplementary material for: Source apportionment, source-specific health risks, and control factors of heavy metals in water bodies of a typical karst basin in southwestern China
Source: PLoS One. 2024 Aug 23;19(8):e0309142. doi: 10.1371/journal.pone.0309142 (PMC11343453; doi:10.1371/journal.pone.0309142)
Supplement: S1 Fig — (PDF) [file pone.0309142.s003.pdf]

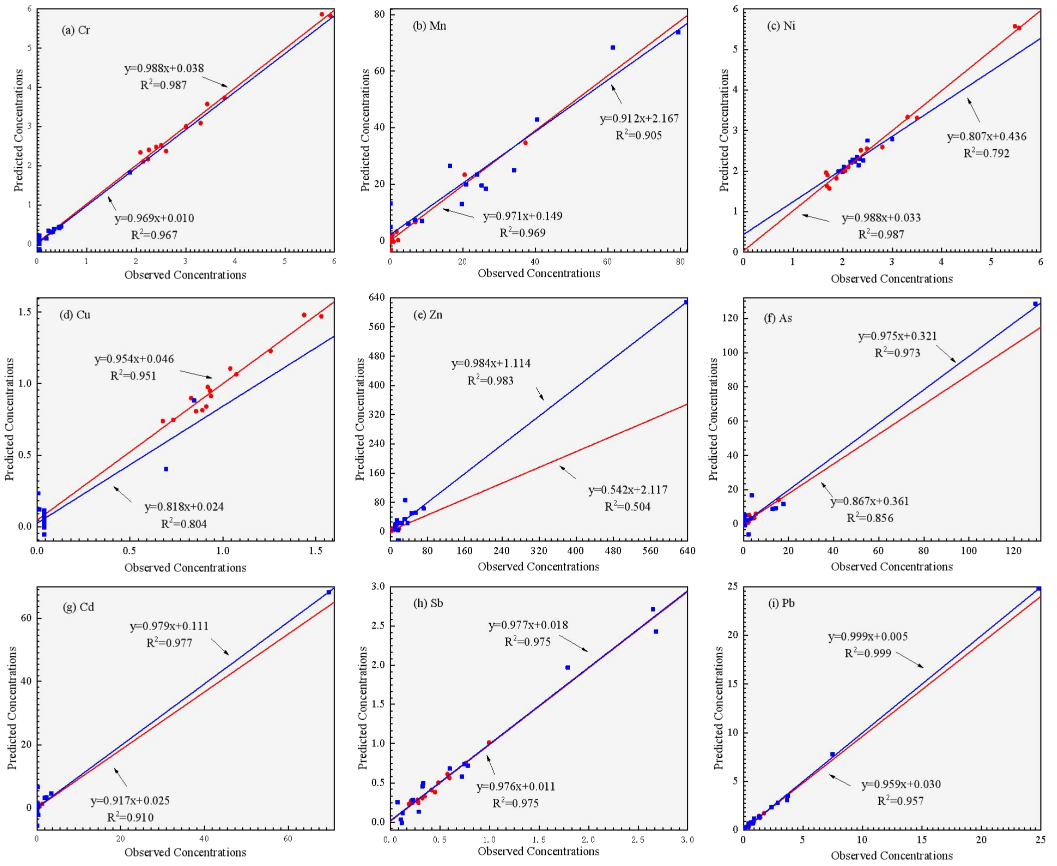

Fig. S1. Scatter plot of predicted and observed heavy metal concentrations using the APCS-MLR model. Red represents the dry season (points and fitted line); blue represents the wet season (points and fitted line).
